# A Window on the Study of Aversive Instrumental Learning: Strains, Performance, Neuroendocrine, and Immunologic Systems

**DOI:** 10.3389/fnbeh.2016.00162

**Published:** 2016-08-24

**Authors:** Caroline C. de Oliveira, Flávia V. Gouveia, Marina C. de Castro, Mayra A. Kuroki, Lennon C. T. dos Santos, Erich T. Fonoff, Manoel J. Teixeira, José P. Otoch, Raquel C. R. Martinez

**Affiliations:** ^1^Laboratory of Neuromodulation and Experimental Pain, Hospital Sirio-LibanesSao Paulo, Brazil; ^2^Division of Functional Neurosurgery, Department of Neurology, School of Medicine, Institute of Psychiatry, University of Sao PauloSao Paulo, Brazil; ^3^Department of Surgery Techniques, School of Medicine, University of Sao PauloSao Paulo, Brazil

**Keywords:** avoidance, aversive instrumental learning, corticosterone, anxiety, elevated plus-maze, immunologic system

## Abstract

The avoidance response is present in pathological anxiety and interferes with normal daily functions. The aim of this article is to shed light on performance markers of active avoidance (AA) using two different rat strains, Sprague-Dawley (SD) and Wistar. Specifically, good and poor performers were evaluated regarding anxiety traits exhibited in the elevated plus maze (EPM) and corticosterone levels and motor activity in the open field test. In addition, the plasma levels of Interleukin-6 (IL-6), Interleukin-1Beta (IL-1beta), Nerve Growth Factor Beta (NGF-beta), Tumor Necrosis Factor-Alpha (TNF-alpha) and cytokine-induced neutrophil chemoattractant 1 (CINC-1) were compared in the good and poor performers to better understand the role of the immunologic system in aversive learning. Behavioral criteria were employed to identify subpopulations of SD and Wistar rats based on their behavioral scores during a two-way AA test. The animals were tested for anxiety-like behavior in the EPM and motor activity in the open-field test. Plasma corticosterone levels were measured at the end of the avoidance test. Cytokine levels of IL-6, IL-1beta, NGF-beta, TNF-alpha, and CINC-1 were measured in the plasma of the Wistar rats. Sixty-six percent of the Wistar rats and 35% of the SD rats exhibited a poor performance. This feature was associated with a decrease in anxiety-like behavior in the EPM. The poor and good performers exhibited lower levels of corticosterone compared with the control animals, which suggests that training alters corticosterone levels, thereby leading to hypocortisolism, independent of the performance. The CINC-1 levels were increased in the poor performers, which reinforces the role of immunologic system activation in learning deficits. Our study provides a better understanding of the complex interactions that underlie neuroimmune consequences and their implications for performance.

## Introduction

Abnormal fear and exaggerated avoidance behavior are present in many anxiety disorders (LeDoux, 2012; Galatzer-Levy et al., 2014). Individual differences in sensitivity to stress and coping behavior in stressful situations are critical in determining vulnerability or resistance to psychopathologies, such as anxiety disorders (Steimer and Driscoll, 2003). Specifically, avoidance behavior is the act of performing a specific motor response to prevent an upcoming aversive event; it involves an active behavioral response to a conditioned threat (Moscarello and LeDoux, 2013). In humans, passive coping styles are associated with increased levels of anxiety (Sheynin et al., 2014). A key characteristic is the marked heterogeneity in which only a minority of individuals develops significant and prolonged symptomatology (Yehuda and LeDoux, 2007).

Animal models may provide information regarding the course and etiology of anxiety disorders and suggest that the susceptibility to develop avoidance behavior is not uniform; rather, susceptibility is determined by sensitivity to specific stimuli or reactions to stimuli experienced during training (Sheynin et al., 2014). One example of these models is the two-way active avoidance (AA) test, which enables an investigation of the transition from fear reactions to instrumental actions (Sidman, 1953). AA may contribute to our understanding of the functional interactions among defense, arousal, reinforcement, motivation and control (Galatzer-Levy et al., 2014).

AA studies have demonstrated that most animals learn the task; however, a significant number of animals (approximately 20%) exhibit a poor performance, and for many years, these animals have been excluded from AA studies (Brush, 1996, 2003). Two different subpopulations, including good and poor avoiders, have been identified in instrumental responses; good avoiders exhibited high AA rates and low freezing following a moderate amount of training, whereas poor avoiders exhibited an opposite pattern (Choi et al., 2010; Lázaro-Muñoz et al., 2010; Martinez et al., 2013). This distinction is important to provide information regarding the normal and abnormal functioning of neurocircuitry, facilitate neurobiology research, and increase the accuracy and translatability of these models (Galatzer-Levy et al., 2014).

Performance anxiety may be explained by an individual inability to suppress the neural networks that underlie fear-elicited reactions. In addition to neurocircuitry, avoidance depends on individual differences (Sheynin et al., 2014). The rate and degree of avoidance acquisition are affected by the strain (Berger and Starzec, 1988; Servatius et al., 2008) and are heritable, trait-like characteristics (Brush et al., 1979). Thus, a number of lines of animals, including Roman Low Avoidance (RLA) and Koltushi Low-Avoidance (KLA), have been bred for their poor performance in AA tasks. It is proposed that anxiety-vulnerable individuals tend to persist with exaggerated avoidance and continue to respond when aversive events no longer occur (Servatius et al., 2008).

Previous studies have focused on understanding the competition in the brain circuits of good and poor avoiders that results in individual differences in instrumental behavior. Selective damage to the central amygdala in poor performers restores the AA performance, which indicates that poor performers had learned but were unable to perform the task (Lázaro-Muñoz et al., 2010). Brain region activation in the amygdala-prefrontal cortex circuits has supported this distinct subpopulation (Martinez et al., 2013; Moscarello and LeDoux, 2013).

These coping strategies are behaviorally and neuroanatomically distinct. In physiological fields, when corticotropin-releasing hormone (CRH), which is released during stressful situations and is a critical mediator of the physiological responses to stress (Dunn and Berridge, 1990), was infused into the central nucleus of the amygdala (CeA), the c-Fos expression increased in the CeA in RLA rats, but not in Roman high avoidance rats (Wiersma et al., 1998). Furthermore, beta-adrenergic receptor blockers effectively treat pathological avoidance behavior in patients with panic disorder and performance anxiety (Ravaris et al., 1991), which suggests that physiological differences may also be responsible for these distinct populations.

Avoidance training sessions induce physiological stress (Coover et al., 1973; Berger and Starzec, 1988). Moreover, a stressful life experience has a positive association with inflammatory diseases (Sternberg, 2001; Elenkov and Chrousos, 2002). Recently, evidence has suggested that impairments in learning, memory and cognitive functions are also induced by inflammatory responses mediated by cytokines (Donzis and Tronson, 2014; Adzovic et al., 2015). Thus, the two-way AA test may induce chronic stress as a result of continuous exposure to a foot shock (King and Hegadoren, 2002), and the stress response would release several inflammatory mediators (Gądek-Michalska et al., 2013; Deak et al., 2015). An inflammatory process produces substantial amounts of proinflamatory cytokines, such as Interleukin-1Beta (IL-1beta), Interleukin-6 (IL-6) and Tumor Necrosis Factor-Alpha (TNF-alpha), which are known to cause cognitive dysfunction (Gądek-Michalska et al., 2013; Donzis and Tronson, 2014; Jing et al., 2015), especially in learning and memory impairment (Farr et al., 2014). Lim et al. (2014) reported that an increase in Nerve Growth Factor (NGF) improved memory formation. Moreover, an increase in IL-6 levels decreased passive avoidance responses (Brunssen et al., 2013). The level of cytokine-induced neutrophil chemoattractant 1 (CINC-1) is correlated with impairments in learning and memory and may represent a biomarker of brain damage (Barichello et al., 2010, 2013). However, few studies have attempted to clarify the correlation between serum levels of cytokines and avoidance performance. The idea of this work was to search for a serum biomarker that could represent the immune activity. Increased cytokine levels have been associated with deficits in memory and neuroplasticity and increases in glial activation (Fiore et al., 2000). Treatment with minocycline (an antibiotic that decreases microglial activation) decreased the production of several proinflammatory cytokines (Choi et al., 2007; Biscaro et al., 2012). Specifically, systemic administration of minocycline attenuated cognitive deficits and increased neurogenesis (Liu et al., 2007; Kohman et al., 2013), suggesting a link between systemic inflammatory biomarkers and learning performance as a consequence of modulation of central immune activity.

We shed light on performance markers for AA using two different rat strains, Sprague-Dawley (SD) and Wistar. We hypothesized that anxiety, different strains and decreases in inflammatory cytokines would facilitate the learning of avoidance behavior. In particular, this research focused on good and poor performers, evaluated the anxiety traits exhibited in an elevated plus maze (EPM) and the motor activity exhibited in the open field test. The plasma levels of corticosterone, IL-6, IL-1beta, NGF-beta, TNF-alpha, and CINC-1 were measured; the controls and good and poor performers were compared to understand the roles of the endocrine and immunologic systems in aversive learning.

## Materials and methods

### Subjects

Male Wistar-derived (*n* = 28) and SD (*n* = 29) rats obtained from the animal facility of the University of Sao Paulo were used as subjects. The animals weighed 230–250 g and were housed in polypropylene cages (40 × 34 × 17 cm) in groups of three under a 12:12 dark/light cycle (lights on at 07:00 h). The room temperature was maintained at 24 ± 1°C, with wood shavings and free access to food and water throughout the experiment. The experiments were performed in compliance with the recommendations of the Brazilian Society of Neuroscience and Behavior, which, in turn, are based on the US National Institutes of Health Guide for the Care and Use of Laboratory Animals. The study was approved by the Ethics Committee on the Use of Animals at Hospital Sirio Libanes (protocol number CEUA 2013/12) and the Medical School of the University of Sao Paulo (protocol number CEP 083/11).

### Study design

The animals were randomized to experimental (SD: *n* = 24 and Wistar: *n* = 23) or control (SD: *n* = 5 and Wistar: *n* = 5) groups. The experimental animals were evaluated in the EPM and the open field test for behavioral analysis. The experimental animals were trained in the two-way AA test for 8 days. The box controls received an equivalent exposure to the box, without the delivery of a foot shock. The EPM, the open field test and the AA test were counterbalanced across rats. After the last day of training, the rats in the experimental and control groups were immediately decapitated for the measurement of serum levels of corticosterone, cytokine and chemokines, see Supplementary Information.

### Apparatus/procedure

#### EMP

Following a habituation period of 5 days, the animals were tested in the EPM. The maze comprised two open arms (50 × 10 cm) crossed at right angles with two opposing arms of the same size, as previously described in detail (Garcia et al., 2005). Two of the opposing arms were enclosed by walls 40 cm high, with the exception of the central section where the arms crossed. The entire apparatus was elevated 50 cm above the floor. To prevent the rats from falling, a rim of Plexiglas (0.5 cm high) surrounded the perimeter of the open arms. The experimental sessions were recorded using a video camera. The rats were gently placed in the central area with the nose facing one of the closed arms and were allowed to explore the maze for 5 min. Before the next rat was tested, the maze was cleaned with a 5% ethanol solution and dried with a cloth.

#### Open field

The animals were tested in the open field, which consisted of a 0.6 m square of dark gray Formica surrounded by 50-cm-high Formica walls. The sessions were recorded with a video camera. Each rat was placed in the center of the open field and allowed to freely explore for 5 min. After each animal completed the test, the open field was cleaned with 5% ethanol and subsequently dried with a dry cloth.

#### Sidman AA

The following day, behavioral training/testing was conducted in 2-way shuttle boxes (Insight Equipment, Ribeirao Preto, Sao Paulo, Brazil) for the experimental and control animals. AA was implemented compared with signaled AA because it is a more difficult protocol that produces a higher percentage of poor avoiders (Choi et al., 2010; Lázaro-Muñoz et al., 2010). The rats received 7 daily 25-min training sessions (excluding weekends). Briefly, shuttling between compartments delayed the delivery of a scrambled foot shock US (1 mA; 0.5 s) by 30 s (R-S interval). In the absence of shuttling, the US delivery occurred every 5 s (S-S interval). The R-S interval shuttles comprised avoidance responses, and the S-S interval shuttles comprised escape responses. All shuttles produced 0.3 s feedback stimuli (house light blink). On day number 8, the good performers, poor performers and additional box control rats were tested in the Sidman avoidance.

#### Corticosterone, cytokines, and chemokines

Blood samples were obtained from the decapitated animals and collected in EDTA-Vacutainer tubes (3 ml trunk blood). The blood was immediately placed on ice and centrifuged. The plasma was separated and stored at −80°C until measurement. For quantification, the plasma corticosterone was determined using a Luminex (Bioplex-200 system (BIO-RAD) and Bio-Plex Manager Software (Bio-Rad Laboratories, Hercules, CA). The samples were measured using a multiplex bead-based immunoassay and were measured in the same assay. The IL-6, IL-1beta, NGF-beta, TNF-alpha, and CINC-1 concentrations were determined in duplicate using specific commercial enzyme-linked immunosorbent assay (ELISA) kits (R&D Systems); Wistar and SD samples were analyzed separately. The ELISA protocol was performed according to the manufacturer's specifications and as described previously (Ballendine et al., 2015).

### Behavioral analyses

A single observer, who was unaware of the groups, recorded the displacements and the other behaviors exhibited in the EPM and open field using the X-Plot Rat Program 2005 Beta 1.0.1. The displacements exhibited by the animals in both apparatuses were recorded. In addition, the frequency and time spent in the following behaviors were measured: (a) head dipping: dipping the head below the level of the maze floor; (b) stretching: the animal stretches to its full length with the forepaws (the hind paws are maintained in the same place) and turns back to the previous position; (c) rearing: partial or total raising on the hind limbs; (d) sniffing: horizontal head movements in any direction, including sniffing of the maze floor and walls; (e) freezing: operationally defined as the total absence of animal movement with the exception of respiration; and (f) grooming: species-specific behavioral sequences, including cleaning of any part of the body surface or fur with the tongue, teeth, and/or forepaws. For Sidman avoidance: shuttling was registered by infrared beams, and the final tests were recorded to DVD for freezing analyses. Freezing was assessed during the first 2 min of the session by an observer blinded to the group specification.

### Statistical analyses

The data are reported as the means ± standard errors of the means (SEMs). The data were analyzed in order to compare differences in performance between strains. Avoidance data were analyzed using an analysis of variance (ANOVA) considering strain, group and session as factors, followed by a Newman-Keuls *post-hoc* test. The box controls were included as a control for the freezing analyses but not for avoidance because, by definition, they could not emit AA responses. The data obtained in the EPM, open field test, and corticosterone assay were analyzed using ANOVA considering strain and group as factors. Significance was set at *P* < 0.05.

## Results

### Avoidance

**Figure 1:**
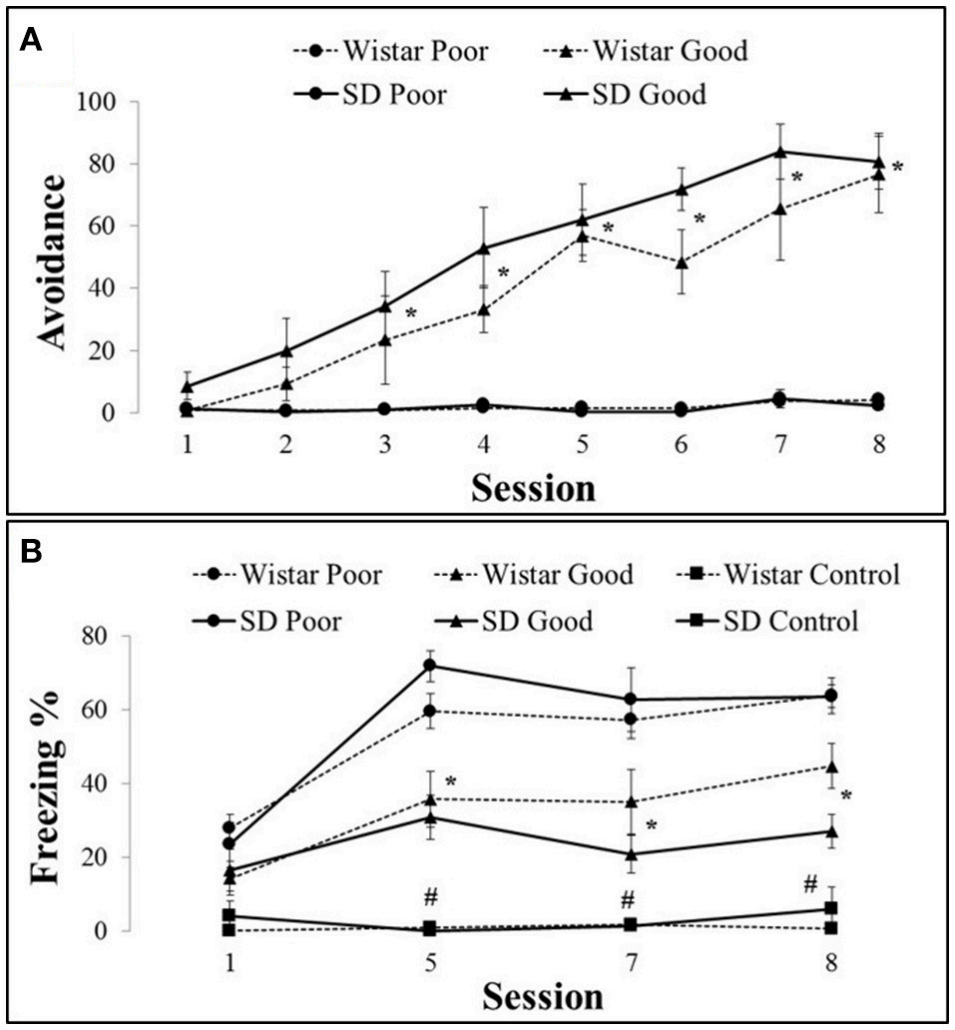
**Number of active avoidance responses in good and poor performers across training sessions with Wistar (A) and Sprague Dawley (B) rats**. Data represent means ± SEMs. ^*^P < 0.05 vs. poor performer, ^#^*P* < 0.05 vs. good and poor performers. SD Good, Sprague Dawley good performers (*n* = 14); SD Poor, Sprague Dawley poor performers (*n* = 10); SD Control, Sprague Dawley control rats (*n* = 5); Wistar Good, Wistar good performers (*n* = 8); Wistar Poor, Wistar poor performers (*n* = 15); Wistar Control, Wistar control rats (*n* = 5).

The training sessions produced a well-characterized behavioral distinction between the good and poor performers. Of the 24 SD and 23 Wistar rats, the good performers represented 66% of the SD (*n* = 14) and 35% of the Wistar (*n* = 8) rats. For the freezing analysis, an additional box control was used (SD *n* = 5, Wistar *n* = 5); however, by definition, these animals never received a shock. The good performers (SD and Wistar) exhibited an increase in avoidance responses (Figure 1A) compared with the poor performers (SD and Wistar) by session 3 [Group × Session, *F*_(14, 588)_ = 7.54, *P* < 0.001]. There were no differences between SD and Wistar considering the same type of performance (i.e., Wistar Poor vs. SD Poor or Wistar Good vs. SD Good; the triple interaction of strain (wistar vs. sd) vs. group (good vs. poor) and session was not significant [*F*_(14, 588)_ = 1.49, *P* > 0.05] nor the interaction between strain and session [*F*_(14, 588)_ = 1.53, *P* > 0.05]. The poor avoiders (SD and Wistar) froze more [Group × Session, *F*_(30, 324)_ = 1.22, *P* < 0.05] than the good performers did by session 5 (Figure 1B). The control group rats froze less compared with the trained animals independent of the lineage. There was no difference between performances considering the strain.

### EPM

**Figure 2:**
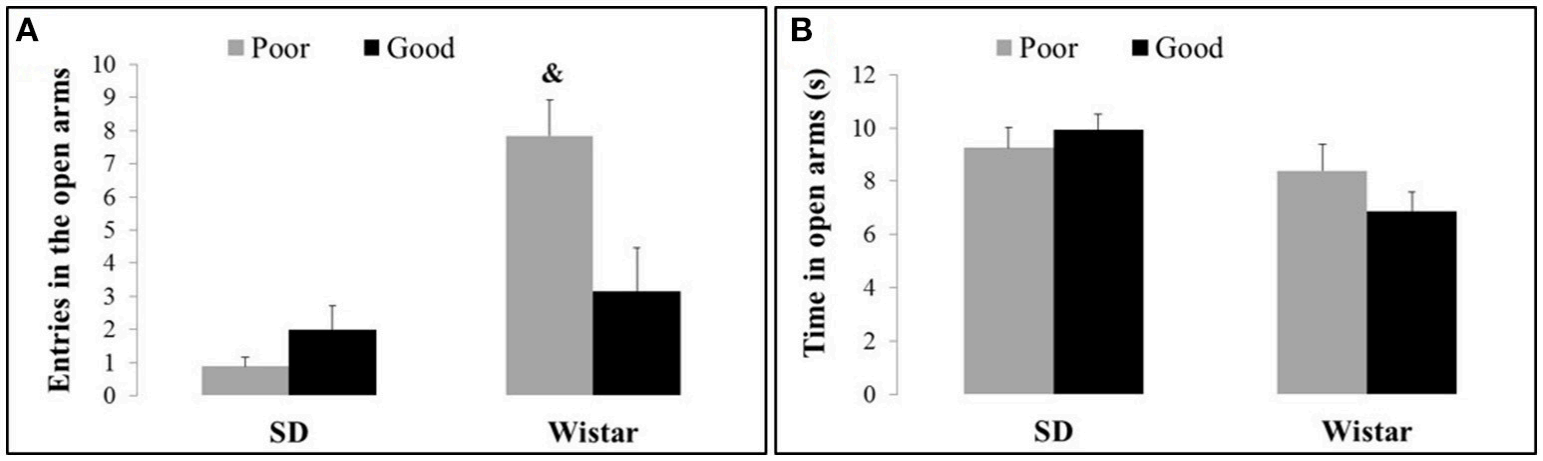
**Entries (A) and time spent (B) in the open arms by good and poor performers in the different strains of Wistar and Sprague Dawley rats**. Bars represent the means, and the vertical lines indicate the SEMs. ^&^*P* < 0.05 vs. all other groups. SD Good, Sprague Dawley good performers (*n* = 14); SD Poor, Sprague Dawley poor performers (*n* = 10); Wistar Good, Wistar good performers (*n* = 8); Wistar Poor, Wistar poor performers (*n* = 15).

Figure 2 shows the entries (Figure 2A) and time spent (Figure 2B) in the open arms exhibited by the good and poor performers considering the different strains of Wistar and SD rats. The Wistar poor performer rats exhibited an increase in the entries in the open arms in comparison with Wistar good performers and SD performers [Strain: *F*_(1, 37)_ = 16.92, *P* < 0.001; Performance: *F*_(1, 37)_ = 3.30, *P* > 0.001 and Strain × Performance *F*_(1, 37)_ = 8.73, *P* < 0.005]. There were no differences in the time spent in the open arms [Strain: *F*_(1, 37)_ = 36.6, *P* < 0.05; Performance: *F*_(1, 37)_ = 1.73, *P* > 0.05 and Strain × Performance *F*_(1, 37)_ = 1.59, *P* > 0.05].

### Open field

**Figure 3:**
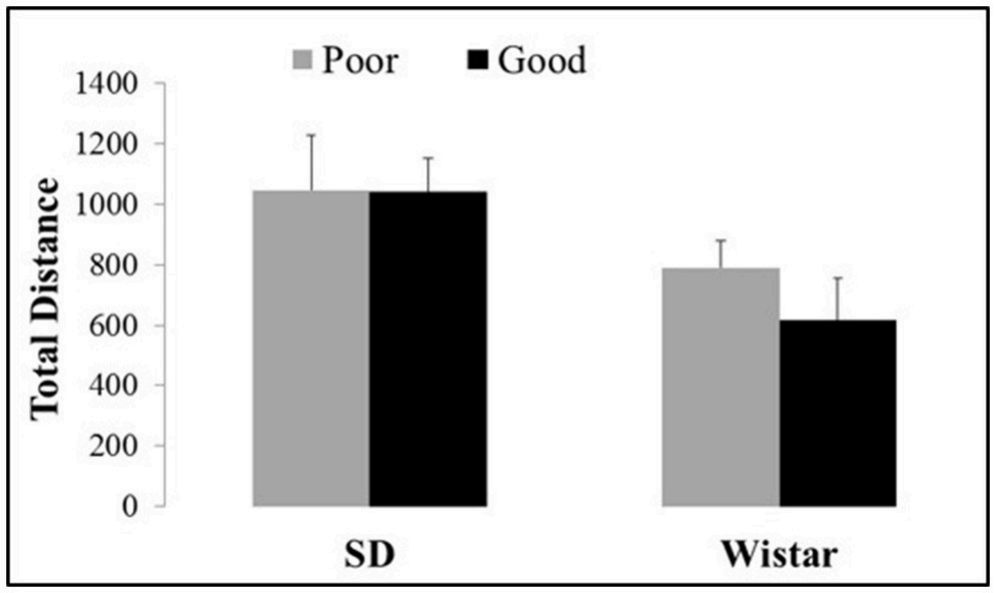
**Total distance traveled in the open field by good and poor performers in different strains of Wistar and Sprague Dawley rats**. Bars represent the means, and the vertical lines indicate the SEMs. SD Good, Sprague Dawley good performers (*n* = 14); SD Poor, Sprague Dawley poor performers (*n* = 10); Wistar Good, Wistar good performers (*n* = 8); Wistar Poor, Wistar poor performers (*n* = 15).

Figure 3 shows the total distance exhibited by the good and poor performers considering the different strains of Wistar and SD rats in the open field. There were no differences in the motor activity between lineages [Strain: *F*_(1, 37)_ = 6.82, *P* < 0.05; Performance: *F*_(1, 37)_ = 0.48, *P* > 0.05 and Strain × Performance *F*_(1, 37)_ = 0.42, *P* > 0.05].

### Corticosterone

**Figure 4:**
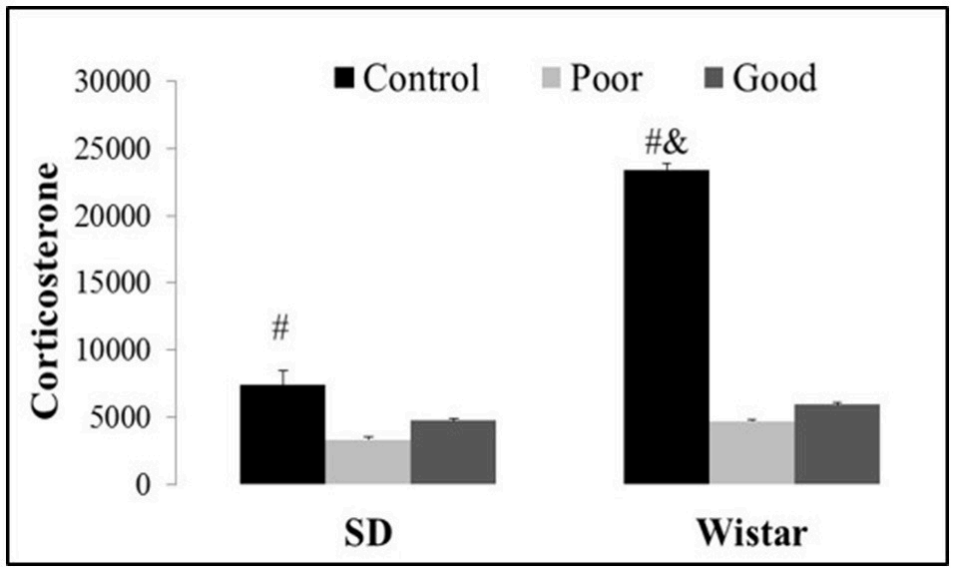
**Effects of AA training on corticosterone levels (pg/dL) in control, good and poor avoiders of two different strains: SD Sprague Dawley and Wistar**. Bars represent the means, and the vertical lines indicate the SEMs. ^#^*P* < 0.05 vs. good and poor performers. ^&^*P* < 0.05 vs. all other groups. SD Good, Sprague Dawley good performers (*n* = 14); SD Poor, Sprague Dawley poor performers (*n* = 10); SD Control, Sprague Dawley control rats (*n* = 5); Wistar Good, Wistar good performers (*n* = 8); Wistar Poor, Wistar poor performers (*n* = 15); Wistar Control, Wistar control rats (*n* = 5).

AA training decreased the corticosterone levels in the good and poor avoiders compared with the corresponding control groups [Strain: *F*_(1, 45)_ = 13.46, *P* < 0.001; Performance: *F*_(1, 45)_ = 15.08, *P* < 0.001 and Strain × Performance *F*_(1, 45)_ = 6.95, *P* < 0.002]. Additionally, Wistar control animals showed an increase in corticosterone levels in comparison with all other groups, as shown in Figure 4.

### Plasma cytokines and chemokines

**Figure 5:**
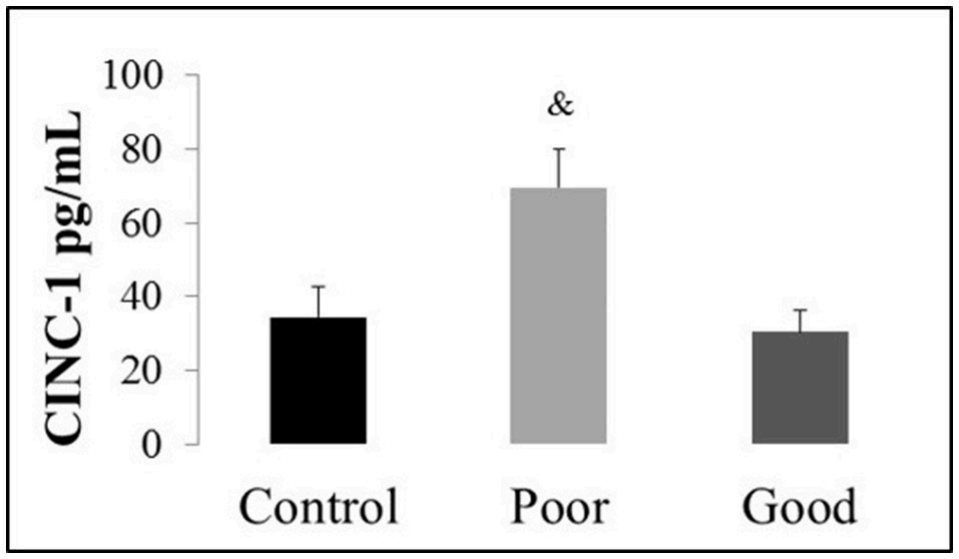
**Plasma concentration of cytokine-induced neutrophil chemoattractant 1 (CINC-1) in the Wistar lineage assessed by enzyme-linked immunosorbent assay**. The data are represented in pg/mL; the bars represent the means, and the vertical lines indicate the SEMs. ^&^*P* < 0.05 vs. Control and Good performers. Wistar Good, Wistar good performers (*n* = 8); Wistar Poor, Wistar poor performers (*n* = 15); Wistar Control, Wistar control rats (*n* = 5).

Using ELISA, we quantified the systemic expression of cytokines (IL-6, IL-1 beta, NGF-beta, and TNF-alpha) and chemokines (CINC-1). The control, poor and good performers had undetectable levels of the circulating cytokines IL-6, IL-1beta, NGF-beta, and TNF-alpha. Specifically, it was not possible to detect IL-6, IL-1beta, NGF-beta, and TNF-alpha in the plasma samples of good and poor performers from Wistar and SD lineages, suggesting that this level of detection is smaller than the detection capability of this kit. For more details regarding the ELISA data, see Supplementary Information 2, which provides an example of IL-1beta detection. Only the CINC-1 levels in the Wistar lineage were detected and exhibited an increase in the poor performers compared with the control and good performers [*F*_(1, 17)_ = 5.18, *P* < 0.02], as illustrated in Figure 5.

## Discussion

First, a behavioral distinction between animals with poor and good performances was demonstrated. The poor performers exhibited substantially reduced responses across the 8 training sessions compared with the good performers. Both groups had a sufficient number of learning trials to acquire the instrumental association; however, the poor performers exhibited little avoidance and tended to express persistent freezing responses. The freezing responses exhibited by the poor performers prevented them from actively avoiding the electric shocks and led them to adopt this form of persistent, passive coping behavior (Steimer and Driscoll, 2003). According to Lázaro-Muñoz et al. (2010) and Choi et al. (2010), the animals had acquired the avoidance response during training; however, they had performance deficits that impaired them from memory expression. The main reason supporting this argument is that the lesion of the CeA was instrumental to AA in the poor performers.

The avoidance task results indicated strain differences in the percentage of good performers (66% in SD vs. 35% in Wistar). Deficits in learning performance across spatial and contextual learning tasks in the Wistar lineage have previously been reported, which suggests that they do not form context parings in an easy and efficient pathway (Keeley et al., 2015). However, the comparison of specific results between Wistar and SD rats are, in some cases, conflicting; this difference may be explained by the differences in the behavioral task. Some authors have reported that the acquisition of the lever presser avoidance is faster and expressed at a higher magnitude in Wistar compared with SD rats (Servatius et al., 2008; Beck et al., 2010; Jiao et al., 2011; Perrotti et al., 2013; Avcu et al., 2014). In contrast, Ferguson and Cada (2004) reported spatial learning and memory deficits in Wistar rats compared with SD rats in the complex maze task. Different strains, as well as the same lineage, including rapid, modal, and slow avoiders and non-avoiders, exhibited a heterogeneous pattern of responses in a signaled AA (Galatzer-Levy et al., 2014). The Wistar lineage has an innate ability to develop extinction-resistant avoidance (Servatius et al., 2008; McAuley et al., 2009; Jiao et al., 2011); this characteristic is important because avoidance is a core symptom of anxiety disorders (American Psychiatric Association., 2013). Thus, the exacerbated anxiety response in Wistar rats may be responsible for decreasing the avoidance response and supports that only 35% of the rats are good performers.

The Wistar lineage is known for its anxiety trait (Servatius et al., 2008; McAuley et al., 2009; Jiao et al., 2011), and our data demonstrated that good Wistar performers exhibited a decrease in the entries in the open arms compared to Wistar poor performers, thus reflecting well-established anxiety-like behaviors (Cruz et al., 1994; Ramos et al., 1997). The current findings suggest that good performers are predisposed to high anxiety because they exhibit more fearful reactions, such as less time spent in the open arms in the EPM. In the same task, good and poor performers of SD lineage exhibited similar EPM behavior. In contrast to our data, Horii et al. (2012) reported that SD high avoiders exhibited high anxiety-like behaviors compared with low avoiders. Furthermore, Steimer and Driscoll (2003) have also identified an increase in the duration and frequency of open arm exploration in roman high avoidance rats compared with the low avoiders. One potential reason for this difference may be the use of inbred strains of high and low avoidance animals, which were originally selected and bred in accordance with their performance in the avoidance task.

The open field test is the classic test used to assess locomotor and exploratory activity (Walsh and Cummins, 1976; Redolat et al., 2009; Overstreet, 2012). The current findings demonstrated that there was no difference in motor learning between performers as indicated by the total distance exhibited in the open field in both lineages. Researchers who have bred lines of Roman and SD rats (Steimer and Driscoll, 2003; Horii et al., 2012) have reported that high performers exhibited an increase in locomotor activity. This increase does not reflect a physical impairment between good and poor performers, which may explain the differences in the avoidance response. The main reason for the poor avoiders not performing instrumental responses is because of malfunctions in the neurocircuitry that involves the prefrontal cortex and amygdala (Wilensky et al., 2000; Choi et al., 2010; Lázaro-Muñoz et al., 2010; Martinez et al., 2013; Moscarello and LeDoux, 2013; McCue et al., 2014; Jiao et al., 2015).

With an emphasis on the neuroendocrine aspect, our data demonstrated that poor and good performers exhibited decreased corticosterone levels after 8 days of the two-way AA compared with the control animals that never received shocks. At first glance, this decrease in corticosterone is contrary to the concept of stress research because evidence predominately indicates an increase in corticosterone levels as a physiological response to stress (King and Hegadoren, 2002; Brush, 2003; Steimer and Driscoll, 2003; Lightman, 2008; Ganella and Kim, 2014; Spiga et al., 2015).

To understand this finding, the mechanisms of cortisol release must be detailed. Its mechanisms involve the sympathetic and hypothalamic-pituitary-adrenal (HPA) systems that are activated when a stimulus is perceived as a stressor (King and Hegadoren, 2002). The activation of the sympathetic system is immediate and results in epinephrine secretion from the adrenal medulla and norepinephrine from peripheral and central sympathetic neurons. HPA axis activation lasts from minutes to hours and results in the release of corticotropin-releasing factor (CRF) from the hypothalamus. This hormone stimulates the release of adrenocorticotropin hormone (ACTH) from the pituitary in the systemic circulation. Finally, ACTH acts on the adrenal cortex to release the glucocorticoid cortisol (Baum and Grunberg, 1997). Based on the HPA axis, several mechanisms may be involved in the development of hypocortisolism. These mechanisms may include reductions in biosynthesis or depletion at several levels of the HPA axis; hypersecretion of CRF and an adaptive down-regulation of the pituitary CRF receptors; an increase in feedback sensitivity of the HPA axis or morphological changes at different levels of the HPA axis. Thus, acute training tasks, such as foot shock, induce the release of adrenal stress hormones, such as corticosterone (Gold and Van Buskirk, 1975). Repeated stress may result in an upregulation of glucocorticoid receptors with an increased sensitivity of the HPA axis to negative feedback inhibition, which may be responsible for decreasing cortisol secretion until the development of chronic hypocortisolism (Boyer, 2000; Heim et al., 2000).

Our hypothesis is that longer duration training (7 days) may be responsible for the elicitation of a repetitive stress, which promotes adaptive effects, such as increasing the number of glucocorticoid receptors and decreasing corticosterone secretion. This may have a protective effect on the nervous system because chronic exposure to high corticosterone impairs new learning in several other aversive and non-aversive contexts (Wolf, 2003). One potential mechanism is that attenuated cortisol leads to an exaggerated catecholaminergic response during a traumatic event, which may result in an over-consolidation of fear (Yehuda et al., 1990; Cabib and Puglisi-Allegra, 1996; Reznikov et al., 2015). Hypocortisolism has been reported in several pathological conditions, including post-traumatic stress disorder, burnout with physical complaints, chronic fatigue syndrome, fibromyalgia, chronic pelvic pain, and asthma (Demitrack et al., 1991; Hellhammer and Wade, 1993; Crofford et al., 1994; Yehuda, 1997; Heim et al., 2000). Supporting our hypothesis, Xu et al. (2015) reported that acute stress, but not chronic stress, increased the plasma corticosterone concentration. Gourley et al. (2009) reported that prior stress or chronic exposure to corticosteroids leads to a decrease in endogenous corticosterone. Furthermore, hypocortisolism has been described in healthy subjects who experience ongoing stress and in animal models of chronic stress (Caplan et al., 1979; Heim et al., 2000).

In terms of avoidance acquisition, the physiological stress levels were maximal in early training and subsequently decreased (Coover et al., 1973; Berger et al., 1981). During early avoidance training (days 1 and 3), high and low avoiders exhibited an increase in corticosterone and ACTH levels compared with basal levels (Akieda-Asai et al., 2011). In this sense, yoked controls (i.e., rats that receive a footshock but that do not have control over shock termination) have also shown an increase in corticosterone levels after 3 days of stress (Berger et al., 1981; Kant et al., 1992) but a decrease in corticosterone levels after 14 days of stress (Kant et al., 1992), supporting the chronic stress effect.

In another avoidance protocol, Rhesus monkeys were required to press a lever to avoid electric shocks, and the cortisol levels constantly decreased to extremely low values (Mason et al., 1968). In humans, Bourne et al. (1968) suggested an association between active coping and decreased adrenal activity, based on data obtained from soldiers who lived in Vietnam camps, had been warned to expect an enemy attack and had to prepare for the event; interestingly, the cortisol metabolite levels decreased with time.

Regarding performance, previous studies have demonstrated that inbred high avoiders exhibited an increase in adrenocorticotropin levels and CRH compared with low avoidance rats (Ohta et al., 1999; Brush, 2003; Akieda-Asai et al., 2011). Data from another strain, the derivation of the Syracuse high and low avoidance, indicated a dissociation of behavioral and endocrine measures in which low avoiders were more anxious but had reduced corticosterone release compared with high avoiders (Brush, 2003). The reason for this discrepancy with our results may be because of inbred rats and the small number of training sessions.

In terms of strain, the Wistar control group exhibited a very high basal level of plasma corticosterone. These data are consistent with the hyper-responsiveness of Wistar rats to stress specifically in neuroendocrine responses, including exaggerated corticosterone, plasma ACTH and norepinephrine levels compared with SD rats (Paré and Redei, 1993; Redei et al., 1994; Fairbanks and Klein, 1996; Gold and Chrousos, 1999; Baum et al., 2006).

In addition to the HPA nervous system, the immune system is impacted by stressful experiences (Deak et al., 2015). Cytokine secretion markedly affected neurotransmission and induced hormonal changes similar to the changes identified following stressor exposure (Gądek-Michalska et al., 2013). Preexisting individual differences in the immune system that may affect social stress have previously been evaluated (Hodes et al., 2014), which reinforces the increasing importance of the role of inflammation in several processes.

Our data indicated undetectable levels of IL-6, IL-1beta, NGF-beta, and TNF-alpha. The classic pro-inflammatory cytokines include IL-1, IL-6, and TNF-alpha; these cytokines modulate the central nervous system during stressor exposure (Deak et al., 2015) and memory processes (Pugh et al., 1998; Elderkin-Thompson et al., 2012). However, the undetectable levels in our plasma samples suggest that these cytokines may be more restricted to the central nervous system structures rather than present in the circulating blood samples. IL-1beta and IL-6 are most commonly found in the brain (Capuron and Miller, 2011). Manipulations in the periphery have been previously shown to increase the expression of IL-1 beta, IL-6, and TNF-alpha in the hippocampus (Datta and Opp, 2008; Cibelli et al., 2010; Burton et al., 2011; Ren et al., 2011). IL-1beta has been identified in key structures, such as the amygdala and the paraventricular nucleus of the hypothalamus, in response to stress (Blandino et al., 2009; Hueston and Deak, 2014). Increased levels of local TNF-alpha n the hippocampus via astrocyte signaling are responsible for cognitive dysfunction (Habbas et al., 2015). Previously, the quantification of NGF was primarily performed with hippocampal (Zheng et al., 2008; Choi et al., 2011; Kim and Oh, 2013) and cortical (Choi et al., 2011) samples.

Other cellular manifestations of neuroimmune activation include the expression of the chemokine CINC-1, the rodent homolog of human IL-8 (Ballendine et al., 2015; Silva et al., 2015), which is responsible for mediating the recruitment of neutrophils (Shibata, 2002; Brochu et al., 2011). CINC-1 was the only detectable chemokine, and one reason may be that neutrophils are the first inflammatory cells to be expressed (Witko-Sarsat et al., 2000). Other reasons may include its role in regulating oligodendrocytes that are responsible for the pattern of white matter tracts in the central nervous system (Vora et al., 2012) or decreased levels of serum CINC-1 that act as neuroprotective agents in blood-brain barrier breakdown (Michalak et al., 2010). In this sense, we suggested that CINC-1 is an important inflammatory tool that could contribute to differential avoidance information processing. There is only one paper (Ballendine et al., 2015) in which the systemic contribution of infiltrating neutrophils to the neuroinflammatory response has been indirectly associated with performance. Specifically, pregnant rats were treated with polyl:C, which increased circulating CINC-1, and the offspring exhibited impairments in recognition memory, visual cues and altered behavioral flexibility in an operant test battery (Ballendine et al., 2015); these findings support our data that increased CINC-1 prejudices performance. To our knowledge, no previous work in the literature has focused on the link between CINC-1 and avoidance or fear conditioning. Therefore, our study provides intriguing results relevant to specific aspects of avoidance and its correlation with systemic CINC-1. There is an inconsistency regarding serum CINC-1 levels in SD and Wistar lineages. The explanation may be that most measurements of serum CINC-1 in rats have utilized Long Evans (Ballendine et al., 2015), Lewis (Brochu et al., 2011) and Wistar (Barichello et al., 2010, 2012, 2013; Camilo et al., 2014; Quinteiro et al., 2014; Sunahara et al., 2014; Teixeira et al., 2014; Fukui et al., 2015; Nolasco et al., 2015) lineages.

The avoidance paradigm has clinical relevance because avoidance is an effective strategy for coping with danger; it is extensively used by patients with fear-related disorders to reduce their exposure to fear- or anxiety-provoking situations. Pathological avoidance is a hallmark of anxiety disorders, and patients are often unable to perform normal daily activities (Mineka and Zinbarg, 2006). Our study provides a better understanding of the complex interactions that underlie these strains and the neuroimmune consequences, as well as their implications for performance. It is important to emphasize that the main contribution of our work is that we have demonstrated for the first time that the plasma level of CINC-1 may be an important biomarker of avoidance impairment. In the future, we expect that regulatory cytokine therapy may represent an effective strategy to regenerate the immune balance and prevent or reverse abnormal fear and exaggerated avoidance behaviors.

## Author contributions

CD and FG both performed the experiments, analyzed the data, and helped to write the paper. MD, MK, and LD performed the experiments and analyzed the data. EF, MT, and JP designed the study and helped to write the paper. RM designed the studied, performed the experiments, analyzed the data and wrote the paper.

## Funding

This research was supported by an FAPESP grant to RM (#11/08575-7) and FAPESP fellowship to CD (#13/03039-5), FG (#13/20602-5), MD (#13/03040-3), and LD (#14/12999-5).

### Conflict of interest statement

The authors declare that the research was conducted in the absence of any commercial or financial relationships that could be construed as a potential conflict of interest.
