# Supplementary material for: A Window on the Study of Aversive Instrumental Learning: Strains, Performance, Neuroendocrine, and Immunologic Systems
Source: Front Behav Neurosci. 2016 Aug 24;10:162. doi: 10.3389/fnbeh.2016.00162 (PMC4995215; doi:10.3389/fnbeh.2016.00162)

**ELISA Data For IL-1beta**

Standard Curve

For each specific cytokine/chemokine, a standard curve was generated that functioned according to the specifications of the ELISA kit. The graph below represents typical data generated when using the ELISA Kits. The standard curve was calculated using a computer-generated 4-PL curve-fit; regression values (r^2^) ranging from 0.98-0.99 are considered reliable. The graph below represents typical data generated when the ELISA Kits were used; specifically, our regression value in the graph below was 0.99966.


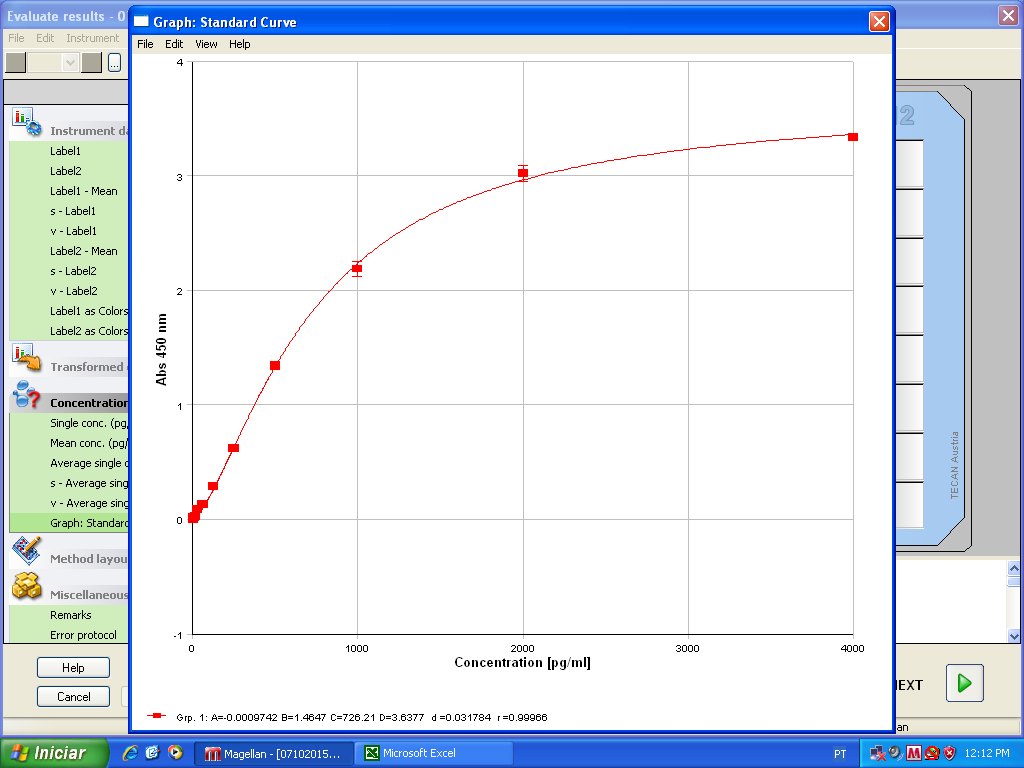


Single Concentration: pg/mL

The green squares correspond to the 11 standard curve points (evaluated in duplicate), the black squares correspond to blank wells, and blue squares correspond to plasma samples (in duplicate) from Wistar and SD lineages. As shown, the level of detection was smaller than the detection capability of this kit. In the Results section, the systemic expression of cytokines were undetectable.


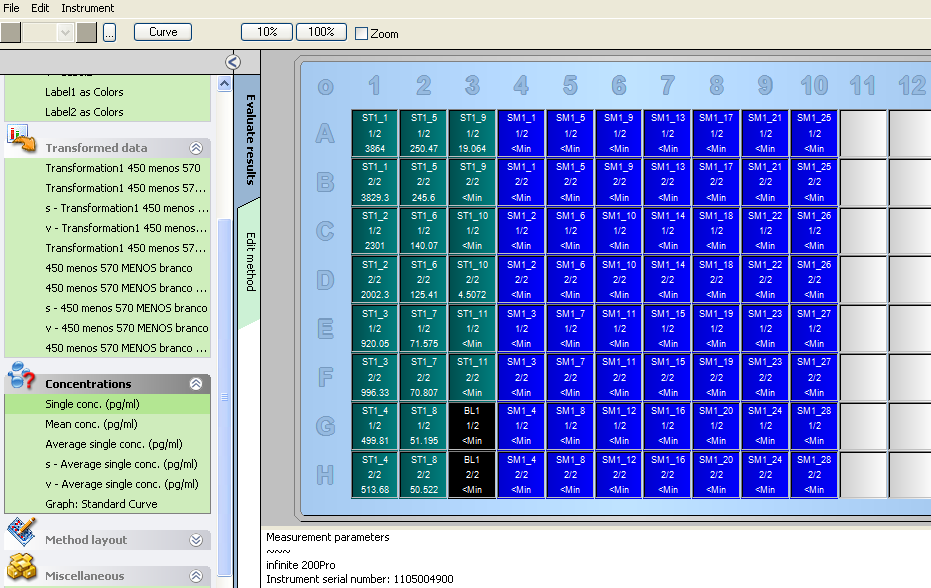

Supplement: Supplementary file 1 [file DataSheet1.DOCX]
