# Supplementary figures and images for: A Window on the Study of Aversive Instrumental Learning: Strains, Performance, Neuroendocrine, and Immunologic Systems
Source: Front Behav Neurosci. 2016 Aug 24;10:162. doi: 10.3389/fnbeh.2016.00162 (PMC4995215; doi:10.3389/fnbeh.2016.00162)

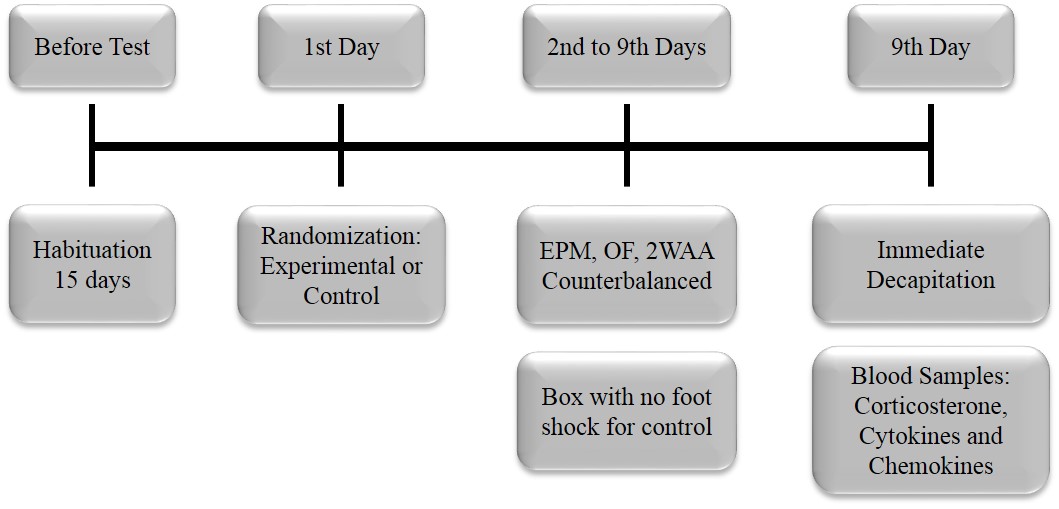

Supplement: Supplementary file 2 [file Image1.JPEG]
